# Clinical significance of EGFR mutation types in lung adenocarcinoma: A multi-centre Korean study

**DOI:** 10.1371/journal.pone.0228925

**Published:** 2020-02-13

**Authors:** Hee-Young Yoon, Jeong-Seon Ryu, Yun Su Sim, Dojin Kim, Sung Yong Lee, Juwhan Choi, Sojung Park, Yon Ju Ryu, Jin Hwa Lee, Jung Hyun Chang

**Affiliations:** 1 Division of Pulmonary and Critical Care Medicine, College of Medicine, Ewha Womans University, Seoul, Republic of Korea; 2 Department of Internal Medicine, Inha University College of Medicine, Incheon, Republic of Korea; 3 Division of Pulmonary, Allergy and Critical Care Medicine, Department of Internal Medicine, Hallym University Kangnam Sacred Heart Hospital, Seoul, Republic of Korea; 4 Division of Allergy and Respiratory Medicine, Soonchunhyang University Bucheon Hospital, Bucheon, Gyeonggi, Republic of Korea; 5 Division of Respiratory, Allergy and Critical Care Medicine, Department of Internal Medicine, Korea University Guro Hospital, Seoul, Republic of Korea; Seoul National University College of Pharmacy, REPUBLIC OF KOREA

## Abstract

Adenocarcinoma is the most common type of non-small cell lung cancer. Some causative genomic alterations in epidermal growth factor receptor (EGFR), including deletions in exon 19 (E19 dels) and a point mutation in E21, are known to have favourable prognoses due to sensitivity to tyrosine kinase inhibitors; however, the prognoses of other uncommon mutations are unclear. This study analysed the clinical significance of EGFR mutation types in lung adenocarcinoma. We retrospectively reviewed 1,020 subjects (mean age: 66.8 years, female: 41.7%) who were diagnosed with advanced lung adenocarcinoma, had EGFR mutation data, and did not undergo surgery from five medical institutes between 2010 and 2016. Subjects were classified according to EGFR mutation status, particularly for exon-specific mutations. EGFR positivity was defined as the presence of mutation and EGFR negativity was defined as wild-type EGFR. EGFR positivity was 38.0%, with the incidence of mutations in E18, E19, E20, and E21 was 3.6%, 51.0%, 3.4%, and 42.0%, respectively. The EGFR positive group survived significantly longer than the negative group (p<0.001), and there was a significant difference in survival among the four EGFR mutation sites (p = 0.003); E19 dels were the only significant factor that lowered mortality (HR: 0.678, p = 0.002), while an E21 mutation was the prognostic factor associated with the most increased mortality (HR: 1.365, p = 0.015). Amongst EGFR positive subjects, the proportion of E19 dels in TKI-responders was significantly higher and that of E21 mutations significantly lower, compared with non-responders. In TKI treatment, mutations in E18 and E20 were not worse factors than the E21 L858R mutation. In conclusion, the presence of EGFR mutations in advanced lung adenocarcinoma can predict a good prognosis; E19 dels prospect to have a better prognosis than other mutations, while an E21 mutation is expected to increase mortality.

## Introduction

Non-small cell lung cancer (NSCLC), particularly in advanced stages, has a very poor prognosis, and conventional systemic chemotherapy only results in an increase of less than one year for overall survival (OS) with a high possibility for toxicity [1–3]. Mutations in epidermal growth factor receptor (EGFR) lead to increased downstream signalling, which promotes cell proliferation, differentiation, and growth [4]. Tyrosine kinase inhibitors (TKIs) that block EGFR-derived signal transduction show excellent efficacy in many patients with EGFR mutations [5–8]. According to the National Comprehensive Cancer Network guidelines, TKIs are currently recommended as first-line treatment for advanced EGFR-mutant NSCLC [9].

EGFR mutations are commonly detected in adenocarcinoma, with higher rates amongst Asians (38.8%–64.0%) than amongst Caucasians (4.9%–17.4%) [10–14]. Almost 90% of all EGFR mutations are deletions in exon 19 (E19 dels) or a leucine to arginine substitution (L858R) in E21, which are generally referred to as “common mutations” [15]. Clinical trials have demonstrated efficacy of TKIs for advanced EGFR-mutant NSCLC patients with these common mutations; however, only a small number (n) of patients with other EGFR mutations were enrolled [5, 6, 16]. Uncommon mutations, including E18, E20 and other complex mutations are relatively rare in NSCLC patients, with a prevalence ranging from 10%–18% [5, 6, 17, 18]. Although some studies have reported sensitivities to TKIs according to EGFR mutation types [19–22], these studies have only focused on differences between the common mutation types. The response to TKIs of NSCLC patients with uncommon mutations, including E18 and E20, and their prognoses has not been fully investigated and previous studies have found conflicting results. In recent studies, uncommon mutations were associated with poorer prognoses compared with common mutations [23–25]. Additionally, there are differences in prognosis among the uncommon mutations; specific uncommon mutations, including G719X in E18, have a good prognosis and were associated with improved TKI responses [26]. However, there have been limited studies comparing the prognoses of common and uncommon EGFR mutations in real-world clinical settings. The purpose of this study was to investigate outcomes of advanced lung adenocarcinomas with regard to EGFR mutation status and TKI treatment responses.

## Methods

### Study population

**Fig 1:**
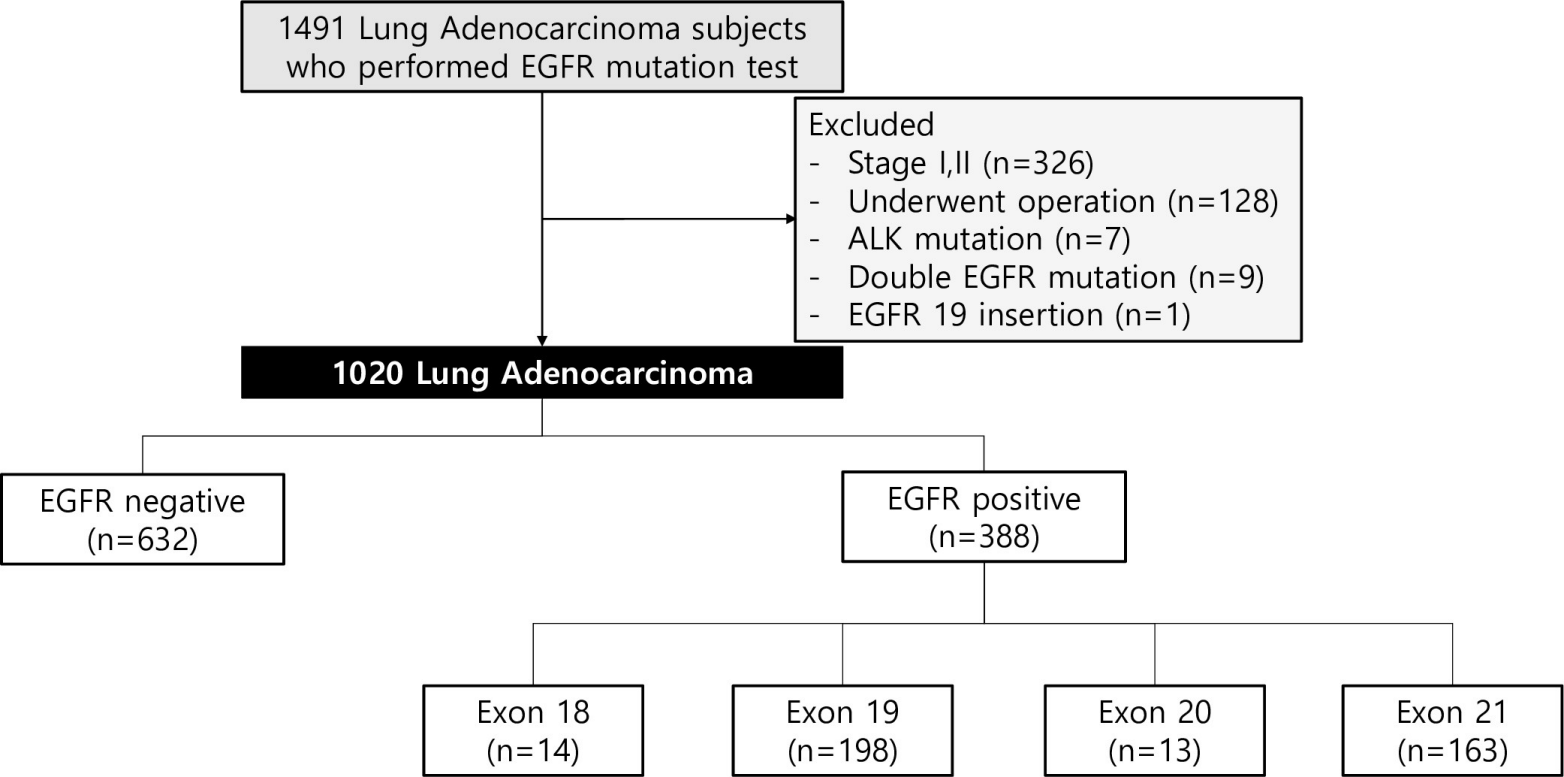
Patient enrolment. EGFR, epidermal growth factor receptor; ALK, anaplastic lymphoma kinase.

Between January 2010 and December 2016, 1491 lung adenocarcinoma subjects who had EGFR sequencing data from five secondary or tertiary medical institutes were screened for our study (Fig 1) (doi.org/10.17504/protocols.io.bahwib7e). Among them, 471 were excluded due to non-advanced stage (n = 326) and surgery (n = 128). Because the frequencies of EGFR duplicate exonal mutations (n = 9), ALK mutation (n = 7) and E19 insertion (n = 1) were relatively low compared with those of other EGFR mutations, these were excluded in this analysis due to concerns of confounding variables in the analysis; thus, 1,020 subjects were included in our study. All subjects were initially classified into EGFR positive and EGFR negative groups. EGFR positivity was defined as the presence of EGFR mutation or insertion/deletion and EGFR negativity was defined as wild-type EGFR. The EGFR positive group was divided according to mutation sites of E18, E19, E20, and E21. Also, EGFR positive lung adenocarcinoma subjects who received more than four TKI cycles were defined as TKI-responders and separately analysed. Informed consent was waived due to the retrospective study design, and the study was approved by the Institutional Review Board of all participating institutes (Ewha Womans University: EUMC 2018-04-043, Hallym University: HKS 2018-04-009, Inha University: 2018-03-017-001, Korean University: 2018GR0013, and Soonchunhyang University: 2018-05-010).

### Study design

Demographics and clinical information of subjects at time of diagnosis were obtained through medical records. The following variables were collected: age, sex, low body mass index (BMI <18.5 kg/m^2^), smoking history, presence of EGFR mutation, stage according to 7th TNM, status of TKI treatment, other treatments (chemotherapy, radiotherapy), OS, forced expiratory volume in 1 second (FEV_1_), and forced vital capacity (FVC) at diagnosis. Variables for Charlson Comorbidity Index (CCI) were also collected to evaluate baseline comorbidities. OS was defined as the time from diagnosis to death or last follow-up and was evaluated for all subjects.

### Tissue preparation and DNA extraction

When tumour tissues were obtained from biopsy, the tissues were fixed with paraffin embedding and stored in the form of cytology slides. When lung cancer was diagnosed in this state, DNA extraction and amplification from tissue proceeded as follows. Tumour cells were scraped with a 26-gauge needle. DNA extraction buffer solution (50 mM Tris buffer, pH 8.3, 1 mM EDTA, pH 8.0, 5% Tween-20, and 100 μg/mL proteinase K) with 10% resin (20–50 μL) was added to the scraped cells. After incubation at 56°C for at least 1 hour, each tube was heated to 100°C for 20 minutes (min) followed by centrifugation at 12,000 rpm for 10 min at 4°C to pellet the debris [27].

#### EGFR mutation testing

EGFR mutation analysis was conducted in two ways according to the facilities and timing of each institution. Between 2010 and 2013, mutation testing was performed using direct sequencing by ISU ABXIS Co Ltd (Seoul, Korea), an independent commercial laboratory. From 2013 to 2016, the peptide nucleic acid (PNA)-mediated PCR clamping method (PNAClamp™ EGFR Mutation Detection Kit, Panagene, Daejeon, Korea) was used to identify EGFR mutation according to the manufacturer’s instructions in the department of pathology at each institute. Complete data analysis and quality control according to each department’s own specific protocols were performed. The target somatic mutations included E19 dels, E21 L858R mutation, E18 G719X mutation, E20 S768I mutation, E20 insertions, E20 T790M mutation, and E21 L861Q mutation. The subtypes of detected mutations are described in S1 Table.

1. *Direct sequencing*. The mutational analyses of EGFR were performed by directional sequencing using polymerase chain reaction (PCR) fragments amplified from genomic DNA. The DNA was amplified with standard PCR technique using each exon-specific primer. PCR products were electrophoresed on 2% agarose gels and were purified with a QIAquick PCR purification kit (QIAGEN, Hilden, Germany). Bidirectional sequencing was performed using the BigDye Terminator v 1.1 kit (Applied Biosystems, Foster City, CA, USA) on an ABI 3130xl DNA analyser (Applied Biosystems) [28].
2. *PNA clamping method*. The principle of the technology is that PNA inhibits amplification of wild-type DNA by hybridizing wild-type sequences, and therefore mutant DNA is dominantly amplified. The amplified mutant type DNA is detected by intercalating dye. PNAClamp analysis was performed using the PNAClamp™ EGFR Mutation Detection Kit (Panagene) following the manual provided by the manufacturer. Briefly, 7 μl of DNA template, 3 μl of each PNA mix, and 10 μl of 2X premix are mixed for a single amplification reaction. The amplification of the mixture (20 μl) was performed in a CFX96 real-time PCR instrument (Bio-Rad, Richmond, CA, USA) with the following thermal program: a pre-incubation at 94°C for 5 min and 40 cycles of amplification (94°C for 30 seconds (s); 70°C for 30 s; 63°C for 30 s; and 72°C for 30 s). Detection of signal of intercalating dye was measured at every step at 63°C. Ct value for the reaction (Sample Ct value) was determined based on the fluorescence values measured at every step at 63°C. If a mutation occurs in a specific codon site, it is not hybridized and amplified so that the Ct value is low. Assessment of the result was determined according to the delta (Δ) Ct value, which was calculated according to each kit manual. ΔCt is the standard Ct value minus the Ct value that was obtained from an unknown sample. The presence of each codon mutation is confirmed by the unique value of ΔCt [29].

### Statistical analysis

All continuous variables were presented as mean ± standard deviation (SD) and categorical variables were presented as number (%). Among EGFR mutation types, continuous variables were analysed by one-way analysis of variance (ANOVA) test or Kruskal-Wallis test and categorical variables were analysed by chi-square test or Fisher’s exact test, when appropriate. Student t-test or Mann-Whitney test were used for comparison of continuous variables between two groups. Bonferroni post hoc test was applied with a p value < 0.01 in variables that showed significant differences in ANOVA for the comparison of five institutes. OS was estimated by using the Kaplan-Meier methods, and difference between groups was assessed by log-rank test. A Cox proportional hazard regression model was used to identify independent factors of OS in subjects with EGFR mutations, presenting hazard ratio (HR) with 95% confidential interval (CI). P-value < 0.05 (two-tailed) was considered statistically significant. All statistical analyses were calculated using SPSS version 24.0 (IBM Corporation, Armonk, NY, USA).

## Results

### Baseline characteristics between EGFR positive and negative subjects

The study cohort included 1,020 subjects; the mean age was 66.8 years, 41.7% were female, 58.7% were never-smokers, and 388 (38.0%) had EGFR mutations (Table 1). The median follow-up period was 13.3 months, and 779 (76.4%) patients died during follow-up. Significant differences by institutes were found in age, smoking status, stage, pulmonary functional parameters, CCI, treatment patterns, and OS (S2 Table). In post hoc analysis of mean OS, there was no statistical difference between any two groups when the p value was cut off at 0.01. Also in univariate and multivariate analysis, OS was not different among institutes (S3 Table).

Subjects with EGFR positive lung adenocarcinoma showed higher rates of female sex, never smokers, and TNM stage IV. They also received more conventional chemotherapy and TKIs compared with EGFR negative subjects. Baseline FEV_1_ was better in EGFR positive subjects than in EGFR negative subjects (Table 1).

**Table 1:** Baseline demographics in EGFR positive and negative lung adenocarcinoma subjects.

	EGFR positive	EGFR negative	Total	p-value
Number(n) (%)	388 (38.0)^1^	632 (62.0)	1,020	
Age	66.2 ± 11.5	67.2 ± 11.4	66.8 ± 11.4^2^	0.190
Sex				<0.001
Male	154 (39.7)	441 (69.8)	595 (58.3)	
Female	234 (60.3)	191 (30.2)	425 (41.7)	
Low BMI (<18.5 kg/m^2^)	28 (7.2)	63 (10.0)	91 (8.9)	0.134
Smoking status (n = 1,017)^3^				<0.001
Ever smoker	97/387 (25.1)	323/630 (51.3)	420/1017 (41.3)	
Never smoker	290/387 (74.9)	307/630 (48.7)	597/1017 (58.7)	
Smoking amount in smoker, pack-years	27.9 ± 18.2	37.1 ± 20.5	35.0 ± 20.3	<0.001
Stage (n = 1012)^3^				0.001
III	43/386 (11.1)	121/626 (19.3)	164/1012 (16.2)	
IV	343/386 (88.9)	505/626 (80.7)	848/1012 (83.8)	
Treatment				
Chemotherapy	321 (82.7)	442 (69.9)	763 (74.8)	<0.001
TKI	284 (73.2)	131 (20.7)	415 (40.7)	<0.001
Radiation therapy	106 (27.3)	166 (26.3)	272 (26.7)	0.712
FEV_1_ (n = 706)^3^	79.2 ± 19.3	76.0 ± 20.3	77.2 ± 20.0	0.041
FVC (n = 706)^3^	77.6 ± 17.7	78.0 ± 17.3	77.9 ± 17.4	0.745
CCI	5.6 ± 2.2	5.9 ± 2.2	5.8 ± 2.2	0.044
Median overall survival, month (95% CI)	22.8 (20.5–25.0)	10.0 (8.9–11.1)	13.8 (12.2–15.5)	<0.001

^1^Number (%);

^2^Mean+/- SD; BMI, body mass index;

^3^Differences in total number are due to missing values. TKI, tyrosine kinase inhibitor; FEV_1_, forced expiratory volume in 1 second; FVC, forced vital capacity; CCI, Charlson comorbidity index; CI, confidence interval.

### Baseline characteristics of EGFR positive subjects

Among the 388 EGFR positive subjects, E19 dels (51.0%) were most common, followed by E21 mutations (42.0%). Mutations in E18 and E20 were identified in 3.6% and 3.4% of EGFR positive cases, respectively (Table 2). There were significant differences among the four EGFR mutation sites in age (67.0 [E18] vs. 64.1 [E19] vs. 66.4 [E20] vs. 68.8 [E21] years, p = 0.002) and rates of never-smokers (71.4% vs. 74.7% vs. 38.5% vs. 78.4%, p = 0.016). Other baseline characteristics were not different among the four groups.

**Table 2:** Baseline demographics according to mutation types among EGFR positive lung adenocarcinoma subjects.

	Exon 18	Exon 19	Exon 20	Exon 21	p-value
Number(n) (%)	14 (3.6)^1^	198 (51.0)	13 (3.4)	163 (42.0)	
Age	67.0 ± 10.1^2^	64.1 ± 11.9	66.4 ± 9.2	68.8 ± 10.7	0.002
Sex					0.102
Male	7 (50.0)	79 (39.9)	9 (69.2)	59 (36.2)	
Female	7 (50.0)	119 (60.1)	4 (30.8)	104 (63.8)	
Low BMI (<18.5 kg/m^2^)	0	17 (8.6)	0	11 (6.7)	0.439
Smoking status (n = 387)^3^					0.016
Ever smoker	4/14 (28.6)	50/198 (25.3)	8/13 (61.5)	35/162 (21.6)	
Never smoker	10/14 (71.4)	148/198 (74.7)	5/13 (38.5)	127/162 (78.4)	
Smoking amount (PY) in smoker	38.5 ± 19.9	27.8 ± 16.9	33.2 ± 29.6	25.7 ± 16.6	0.475
Stage (n = 386)^3^					0.941
III	1/13 (7.7)	22/197 (11.2)	2/13 (15.4)	18/163 (11.0)	
IV	12/13 (92.3)	175/197 (88.8)	11/13 (84.6)	145/163 (89.0)	
Treatment					
Chemotherapy	11 (78.6)	157 (79.3)	12 (92.3)	141 (86.5)	0.234
TKI	9 (64.3)	140 (70.7)	8 (61.5)	127 (77.9)	0.268
Radiation therapy	4 (28.6)	56 (28.3)	4 (30.8)	42 (25.8)	0.944
FEV_1_ (n = 269)^3^	77.5 ± 25.8	79.0 ± 17.8	80.1 ± 16.4	79.6 ± 21.1	0.982
FVC (n = 269)^3^	79.9 ± 23.5	76.9 ± 16.8	83.0 ± 18.6	77.9 ± 18.3	0.763
CCI	5.7 ± 2.2	5.5 ± 2.2	4.8 ± 1.6	5.7 ± 2.2	0.440
Median overall survival, month (95% CI)	17.2 (10.5–24.0)	29.9 (22.4–37.4)	19.1 (8.3–30.0)	20.6 (16.9–24.3)	0.003

^1^ Number (%);

^2^Mean+/- SD;

^3^Differences in total number are due to missing values. BMI, body mass index; PY, pack-years; TKI, tyrosine kinase inhibitor; FEV_1_, forced expiratory volume in 1 second; FVC, forced vital capacity; CCI, Charlson comorbidity index; CI, confidence interval.

Of the 284 subjects who received TKI, 250 (88.0%) were TKI-responders. TKI-responders were younger; the TKI- responder group had a significantly higher proportion of E19 dels and a significantly lower rate of E21 mutations than the TKI non-responder (Table 3).

**Table 3:** Comparison of baseline characteristics between TKI-responders and non-responders in EGFR positive lung adenocarcinoma.

	Responder^1^	Non-responder	Total	p-value
Number(n) (%)	250 (88.0)^2^	34 (12.0)	284	
Age	66.5 ± 10.7^3^	72.0 ± 10.8	67.2 ± 10.8	0.006
Sex				0.653
Male	93 (37.2)	14 (41.2)	107 (37.7)	
Female	157 (62.8)	20 (58.8)	177 (62.3)	
Low BMI (<18.5 kg/m^2^)	20 (8.0)	3 (8.8)	23 (8.1)	0.745
Smoking status (n = 283)^4^				0.924
Ever smoker	64/249 (25.7)	9/34 (26.5)	73/283 (25.7)	
Never smoker	185/249 (74.3)	25/34 (73.5)	210/283 (74.2)	
Smoking amount (PY) in smoker	28.1 ± 19.1	30.3 ± 21.4	28.3 ± 19.2	0.765
Stage				0.054
III	25 (10.0)	0 (0.0)	25 (8.8)	
IV	225 (90.0)	34 (100.0)	259 (91.2)	
FEV_1_ (n = 208)^4^	79.7 ± 19.9	77.5 ± 19.7	79.4 ± 19.8	0.609
FVC (n = 208)^4^	78.4 ± 17.8	78.4 ± 16.2	78.4 ± 17.6	1.000
CCI	5.6 ± 2.1	6.2 ± 2.4	5.7 ± 2.2	0.138
Mutation type				
E18	9 (3.6)	0	9 (3.2)	0.606
E19	129 (51.6)	11 (32.4)	140 (49.3)	0.035
E20	6 (2.4)	2 (5.9)	8 (2.8)	0.246
E21	106 (42.4)	21 (61.8)	127 (44.7)	0.033
Median overall survival, month (95% CI)	28.2 (23.6–32.8)	11.2 (NO-24.0)	25.1 (20.9–29.4)	<0.001

^1^TKI responder was defined as a patient who received more than 4 cycles of TKI;

^2^Number (%);

^3^Mean+/- SD;

^4^Differences in total number are due to missing values. TKI, tyrosine kinase inhibitor; BMI, body mass index; PY, pack-years; FEV_1_, forced expiratory volume in 1 second; FVC, forced vital capacity; CCI, Charlson comorbidity index; E, Exon; NO, not obtained; CI, confidence interval.

### Comparison of survival curves

**Fig 2:**
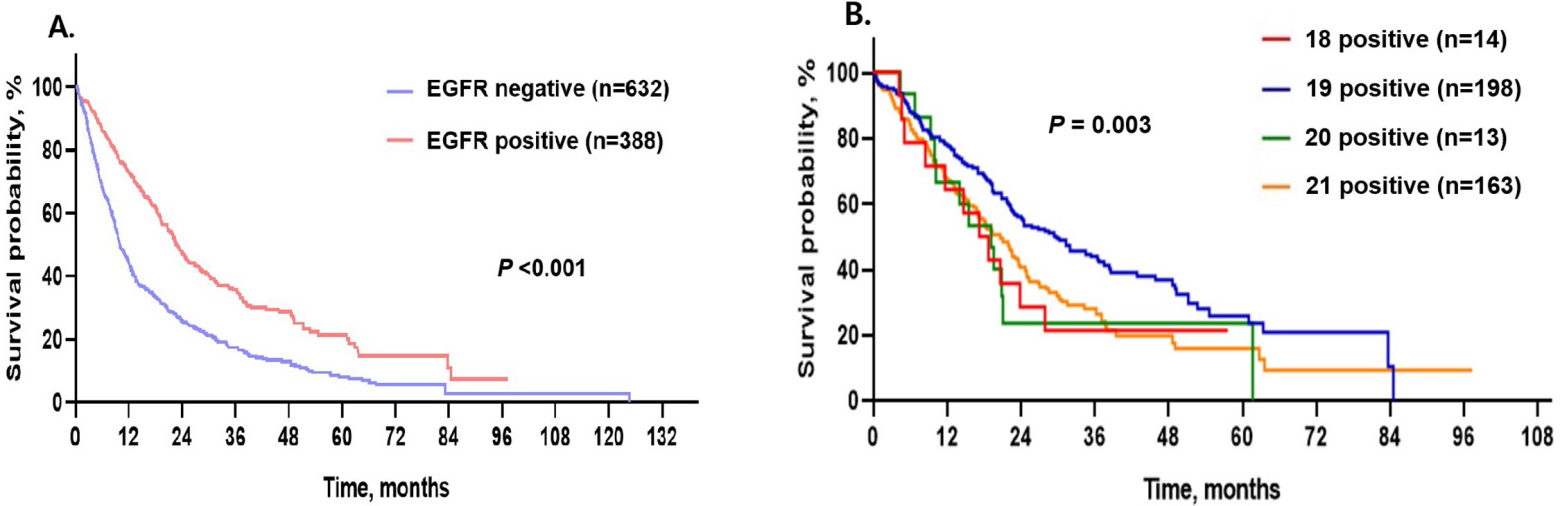
Survival curves in subjects with advanced lung adenocarcinoma. A. Comparison of survival curves between EGFR positive and negative lung adenocarcinoma. B. Comparison of survival curves among mutation types in EGFR positive lung adenocarcinoma.

Amongst all subjects, the median OS was 13.8 months (95% CI: 12.2–15.5 months). EGFR positive subjects had better median OS (22.8 months, 95% CI: 20.5–25.0 months) than EGFR negative subjects (10.0 months, 95% CI: 8.9–11.1 months, p < 0.001; Table 1 and Fig 2A).

Within EGFR positive subjects, there were also significant differences in survival curves among the four EGFR mutation sites (Table 2; p = 0.003). Subjects with E19 dels had the best median OS (29.9 months, 95% CI: 22.4–37.4 months), while E18 mutations were associated with the worst median OS (17.2 months, 95% CI: 10.5–24.0 months). The median OS of subjects with E20 and E21 mutations were 19.1 months (95% CI: 8.3–30.0 months) and 20.6 months (95% CI: 16.9–24.3 months), respectively (Table 2 and Fig 2B).

#### Comparison of survival curves in subjects with TKI treatment

**Fig 3:**
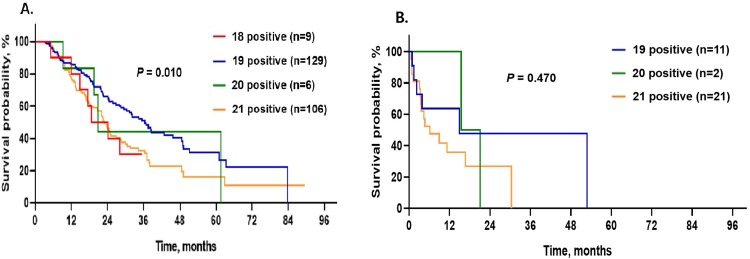
Survival curves in subjects with EGFR positive lung adenocarcinoma according to TKI response. A. Comparison of survival curves among EGFR mutation types in TKI-responders. B. Comparison of survival curves among EGFR mutation types in TKI non-responders.

The median OS of TKI-responders was 28.2 months (95% CI: 23.6–32.8 months), while that of TKI non-responders was 11.2 months (95% CI: not obtained–24.0 months) (Table 3). Amongst TKI-responders, subjects with E19 dels had significantly longer median OS (36.4 months, 95% CI: 30.5–42.3 months, p = 0.010) than subjects with E18 (18.6 months, 95% CI: 14.7–22.5 months), E20 (20.8 months, 95% CI: 18.2–23.3 months), and E21 (22.9 months, 95% CI: 19.6–26.2 months) mutations (Fig 3A). There were no significant differences in OS between TKI non-responders with regard to mutation site (p = 0.470; Fig 3B). Amongst all subjects who were treated with TKI, there were no significant differences in median OS between subjects with E18 (18.6 months, 95% CI: 14.7–22.5 months) and E21 (22.0 months, 95% CI, 17.6–26.4 months, p = 0.818) mutations, nor between subjects with E20 (20.8 months, 95% CI, 18.9–22.6 months) and E21 (22.0 months, 95% CI, 17.6–26.4 months, p = 0.927) mutations.

### Risk factors for mortality in EGFR positive subjects

E19 dels and an E21 mutation were inversely associated with mortality compared with EGFR negative subjects, while E18 and E20 mutations did not show statistically significant associations with mortality (Table 4). In EGFR positive subjects, E19 dels, an E21 mutation, age, low BMI, smoking status, stage, CCI, and use of TKI were associated with mortality in univariate analysis. In a multivariate analysis model including E19 dels, age (HR: 1.029, 95% CI: 1.017–1.041), low BMI (HR: 1,729, 95% CI: 1.108–2.700), stage IV (HR: 2.246, 95% CI: 1.426–3.536), use of TKI (HR: 0.533, 95% CI: 0.405–0.702) as well as E19 dels (HR: 0.678, 95% CI: 0.527–0.872) were significant factors for mortality. Conversely, a multivariate analysis model including an E21 mutation revealed that age (HR: 1.030, 95% CI: 1.018–1.042), low BMI (HR, 1,658, 95% CI: 1.065–2.582), stage IV (HR:2.296, 95% CI: 1.458–3.615), use of TKI (HR: 0.527, 95% CI: 0.400–0.695) as well as an E21 mutation (HR: 1,365, 95% CI: 1.063–1.754) were significant factors for mortality.

**Table 4:** Univariate and multivariate Cox proportional hazard analysis for mortality in EGFR positive lung adenocarcinoma subjects.

	Univariate		Multivariate model-1		Multivariate model-2	
	HR (95% CI)	p-value	HR (95% CI)	p-value	HR (95% CI)	p-value
Mutated exon^1^						
18	1.398 (0.763–2.559)	0.278				
19	0.638 (0.498–0.818)	<0.001	0.678 (0.527–0.872)	0.002		
20	1.648 (0.899–3.022)	0.106				
21	1.408 (1.099–1.804)	0.007			1.365 (1.063–1.754)	0.015
Age	1.025 (1.013–1.037)	<0.001	1.029 (1.017–1.041)	<0.001	1.030 (1.018–1.042)	<0.001
Female	0.837 (0.651–1.078)	0.168				
Low BMI (<18.5 kg/m^2^)	1.591 (1.025–2.468)	0.038	1.729 (1.108–2.700)	0.016	1.658 (1.065–2.582)	0.025
Never smoker	1.086 (0.811–1.456)	0.579				
Stage IV	1.916 (1.225–2.999)	0.004	2.246 (1.426–3.536)	<0.001	2.296 (1.458–3.615)	<0.001
CCI	1.110 (1.050–1.173)	<0.001				
Chemotherapy	0.758 (0.550–1.043)	0.089				
TKI	0.674 (0.515–0.883)	0.004	0.533 (0.405–0.702)	<0.001	0.527 (0.400–0.695)	<0.001
Radiation therapy	1.120 (0.856–1.464)	0.408				

^1^Mutations on a specific exon were compared with mutations on the rest of exons. BMI, body mass index; CCI, Charlson comorbidity index; TKI, tyrosine kinase inhibitor.

### Comparison between E19 dels and E18/20/21 mutations in EGFR positive subjects

We also compared E19 dels to E18/20/21 mutations in terms of baseline demographics and a survival curve in EGFR positive subjects. Subjects with E19 dels were younger than subjects with other mutations (64.1 ± 11.9 [E19 dels] vs. 68.5 ± 10.5 [E18/20/21 mutations] years; p < 0.001). In addition, subjects with E19 dels had better median OS (29.9 months, 95% CI: 22.4–37.4 months) than subjects with E18/20/21 mutations (19.2 months, 95% CI: 16.1–22.4 months, p < 0.001) (S4 Table and S1 Fig).

## Discussion

This study first investigated clinical and prognostic features of advanced adenocarcinoma according to EGFR mutation status in Korean patients, particularly for specific EGFR mutation types (mutations in E18, E19, E20, and E21). Similar studies have previously examined common and uncommon EGFR mutations with limited prognostic comparisons for NSCLC [23, 25, 30]. Our study identified that E19 dels were a predictor for good prognosis in EGFR positive lung cancer, similar to previous results [31–35]; conversely, an E21 mutation significantly increased mortality. These results were maintained when we analysed only TKI-responders. Mutations in E18 and E20 were not worse factors than an E21 mutation in lung adenocarcinoma.

In this study, EGFR-mutant lung adenocarcinoma patients had better survival rate than patients with wild-type EGFR. It is well known that TKI treatment leads to a dramatically improved prognosis in patients with EGFR positive lung cancer [5–8]. In our cohort, the EGFR positive group had a higher prevalence of stage IV lung cancer, but a significantly better prognosis, which was probably related to higher rates of TKI treatment. This result is supported by the fact that 73.2% of EGFR positive patients received TKI treatment in our study. Additionally, never-smoker lung cancer, especially of adenocarcinoma histology, was highly associated with female sex of East Asian ethnicity and higher prevalence of genomic alterations [36], which was in accordance with our findings.

Our study demonstrated significant differences in survival curves amongst the four EGFR mutation sites, with a good prognosis in patients with E19 dels and relatively adverse prognosis in patients with an E21 mutation. The fact that TKI is more beneficial in patients with E19 dels than in those with an E21 mutation with regard to OS and progression-free survival (PFS) has already been reported in a number of studies [31–34]. In a meta-analysis by Zhang et al. [34], six clinical trials documented that patients with E19 dels were associated with a better prognosis than patients with an E21 L858R mutation (HR of E19 dels to E21 L858R mutation:  0.59, 95% CI: 0.38–0.92, p  =  0.019). These findings were in line with our results.

According to our study, mutations in E18 and E20 did not significantly influence prognosis compared with previous studies on the prognosis of uncommon mutations [22, 24, 37]. Due to the low incidence of E18 and E20 mutations, limited studies are available on the prognosis of patients with uncommon EGFR mutations. Sutiman et al. [37] found significant differences in PFS (p < 0.001) between patients with different exon mutations, with the best median PFS among E19 dels (n = 202, 10.9 months) and the worst among E20 mutations (n = 9, 1.2 months), which serve as independent predictors for PFS (HR: 16.0, 95% CI: 6.88–37.1, p < 0.001). However, the authors also reported no significant differences in OS curves among EGFR mutation types. Beau-Faller et al. [22] found that OS of patients with E18 mutations (n = 18, median: 22 months) was better than that of patients with E20 mutations (n = 25, median: 9.5 month) in patients with TKI treatment. Chiu et al. [24] reported that patients with uncommon mutations, including G719X (n = 78), L861Q (n = 57), and S768I (n = 7), benefited from TKI treatment, but with lower efficacy than those with common mutations (median OS: 24 [uncommon] vs. 29.7 [common] months, p = 0.005). The study by Yang et al. [21] that was based on afatinib phase 2 and 3 clinical trials revealed that patients with a point mutation or duplications in E18–21 had better PFS and OS than those with E20 insertions or T790M mutations. This discordance might be attributable to a small sample size or different definitions of the “uncommon group.” Thus, a single prognostic evaluation under the umbrella of “uncommon mutation” to represent several exonal variations cannot be justified because prognosis is different for each type. Krawczyk et al. [25] found that among 180 NSCLC patients who were treated with 1st or 2nd line TKIs, patients with uncommon mutations (n = 13) had shorter PFS (median: 5 [uncommon] vs. 10 [common] months, p = 0.005), but similar median OS (26 vs. 22 months, p = 0.251) compared with patients with common mutations of E19 dels and a L858R point mutation. Considering the different TKI sensitivity between patients with E19 dels and an E21 mutation, it might be inappropriate to simply divide into common and uncommon mutations when evaluating OS. In our study, the prognosis of uncommon mutations in adenocarcinoma treated with TKIs was worse than that of E19 dels and similar to that of an E21 mutation. When an uncommon EGFR mutation is detected in lung adenocarcinoma, TKI treatment should be actively considered. Comparison of E19 dels to E18/20/21 mutations showed younger age and better OS in patients with E19 dels. The prognostic differences in EGFR mutations may be due to functional differences between deletions and other kinds of mutations. The functional loss due to exonal deletion in cancer cells will be a good prognostic factor, compared with other types of mutation, but further research is needed at this point.

This study had several limitations. First, although statistically corrected for the effects of variables, bias may exist from two kinds of EGFR test methods and a long observational study. Second, survival differences among institutes existed, but Bonferroni post hoc test showed no significance, and the differences of OS by institutes were influenced by age, sex, BMI, stage, CCI, and use of TKI. Third, early in the study, mutations other than EGFR were not routinely checked, so the rate of ALK mutations was relatively low. Fourth, the number of cases with E18 or E20 mutations was relatively small and can create bias in the analysis. Despite these limitations, the strength of this paper is that the prognosis according to EGFR exon mutational status was targeted to lung adenocarcinoma, a representative type for EGFR mutations, and exon locations of EGFR mutations that included specific types were focused to help assess clinical response to TKIs and their prognosis.

## Conclusions

We found that EGFR positive advanced adenocarcinoma patients had better OS compared with EGFR negative due to response to TKI treatment. In lung adenocarcinoma with EGFR positivity, E19 dels predicts a good prognosis, while an E21 mutation is expected to increase mortality. In TKI treatment, mutations in E18 and E20 were not worse factors than the E21 L858R mutation, unlike previous reports. Further large-scale prospective studies will be needed to verify these results.

## Supporting information

S1 Table EGFR mutation subtypes. (DOCX)

S2 Table Demographic data from 5 medical institutes in lung adenocarcinoma subjects. (DOCX)

S3 Table Univariate and multivariate Cox proportional hazard analysis for mortality in lung adenocarcinoma subjects. (DOCX)

S4 Table Comparison of baseline characteristics between exon 19 deletions and exon 18/20/21 mutations in EGFR positive lung adenocarcinoma subjects. (DOCX)

S1 Fig Comparison of survival curves between exon 19 deletions and exon 18/20/21 mutations in EGFR positive lung adenocarcinoma. (TIF)

S1 Data Data file. (XLSX)
