# Supplementary material for: Clinical significance of EGFR mutation types in lung adenocarcinoma: A multi-centre Korean study
Source: PLoS One. 2020 Feb 13;15(2):e0228925. doi: 10.1371/journal.pone.0228925 (PMC7018076; doi:10.1371/journal.pone.0228925)
Supplement: S1 Table — (DOCX) [file pone.0228925.s001.docx]

**S1 Table.** EGFR mutation subtypes

| **Exon** | **Type** | **Amino acid change** | **Base change** | **No^1^** | **No of subtype^2^** | |
| --- | --- | --- | --- | --- | --- | --- |
| 18 | G719X | G719A | c.2156G>C | 13 | | 0 |
|  |  | G719S | c.2155G>A |  |  | 1 |
|  |  | G719C | c.2155G>T |  |  | 0 |
|  | unspecific |  |  | 1 | | 1 |
| 19 | e19del | p.E746_A750del | c.2235_2249 del 15 | 41 | | 14 |
|  |  | p.E746_T751>A | c.2237_2251 del 15 |  |  | 2 |
|  |  | p.E746_A750del | c.2236_2250 del 15 |  |  | 12 |
|  |  | p.L747_T751>Q | c.2238_2252>GCA |  |  | 1 |
|  |  | p.L747_T751del | c.2239_2253 del 15 |  |  | 1 |
|  |  | p.L747_S752del | c.2239_2256 del 18 |  |  | 1 |
|  |  | p.L747_A750>P | c.2239_2248 TTAAGAGAAG>C |  |  | 1 |
|  |  | p.L747_753>S | c.2240_2257 del 18 |  |  | 2 |
|  |  | p.L747_T751del | c.2240_2254 del 15 |  |  | 5 |
|  |  | p.L747 A755T | c.2240_2263delinsCAACAT |  |  | 1 |
|  |  | p.S752_I759delSPKANKEI | c.2253-2276 del 24 |  |  | 1 |
|  |  | 19 del unspecific |  | 157 | | 157 |
| 20 | S768I | p.V769_D770insASV | c.2307_2308 ins9 | 3 | | 2 |
|  |  | p.Ser768_Asp770dup | c.2302_2310 dupAGCGTGGAC |  |  | 1 |
|  | E20 Ins.3dup | p.G773_V774insH | c.2319_2320 insCAC | 8 | | 3 |
|  | E20 Ins.3 | p.D770_N771insG | c.2310_2311 insGGT |  |  | 1 |
|  | E20 Ins. unspecific |  |  |  |  | 4 |
|  | Q787Q | p.Gln787 | c.2361G>A | 1 | | 1 |
|  | unspecific |  |  | 1 | | 1 |
| 21 | L858R | p.L858R | c.2573T>G | 92 | | 91 |
|  | L861Q | p.L861Q | c.2582T>A |  |  | 1 |
|  | unspecific |  |  | 71 | | 71 |

**^1^**number of exon mutation type, **^2^**number of specific identified amino acid change.
