# Supplementary material for: Clinical significance of EGFR mutation types in lung adenocarcinoma: A multi-centre Korean study
Source: PLoS One. 2020 Feb 13;15(2):e0228925. doi: 10.1371/journal.pone.0228925 (PMC7018076; doi:10.1371/journal.pone.0228925)
Supplement: S2 Table — (DOCX) [file pone.0228925.s002.docx]

| S2 Table. Demographic data from 5 medical institutes in lung adenocarcinoma subjects | | | | | | | |
| --- | --- | --- | --- | --- | --- | --- | --- |
| Institute | **1** | | **2** | **3** | **4** | **5** | **p-value** |
| Number (n) | 233 | | 222 | 134 | 176 | 255 |  |
| EGFR status |  | |  |  |  |  | 0.277 |
| Negative | 143 (61.4)^1^ | | 128 (57.7) | 86 (64.2) | 120 (68.2) | 155 (60.8) |  |
| Positive | 90 (38.6) | | 94 (42.3) | 48 (35.8) | 56 (31.8) | 100 (39.2) |  |
| Mutation type |  | |  |  |  |  |  |
| Exon 18 | 1 (0.4) | | 3 (1.4) | 6 (4.5) | 2 (1.1) | 2 (0.8) | 0.019 |
| Exon 19 | 44 (18.9) | | 44 (19.8) | 25 (18.7) | 34 (19.3) | 51 (20.0) | 0.997 |
| Exon 20 | 6 (2.6) | | 4 (1.8) | 1 (0.7) | 1 (0.6) | 1 (0.4) | 0.185 |
| Exon 21 | 39 (16.7) | | 43 (19.4) | 16 (11.9) | 19 (10.8) | 46 (18.0) | 0.093 |
| Age | 69.0 ± 11.5**^2^** | | 65.5 ± 11.1 | 66.9 ± 11.7 | 65.6 ± 12.3 | 66.8 ± 10.7 | 0.008 |
| Sex |  | |  |  |  |  | 0.955 |
| Male | 133 (57.1) | | 130 (58.6) | 82 (61.2) | 101 (57.4) | 149 (58.4) |  |
| Female | 100 (42.9) | | 92 (41.4) | 52 (38.8) | 75 (42.6) | 106 (41.6) |  |
| BMI<18.5 Kg/m^2^ | 22 (9.4) | | 21 (9.5) | 18 (13.4) | 12 (6.8) | 18 (7.1) | 0.234 |
| Smoking status (n=1017)^3^ | | |  |  |  |  | <0.001 |
| Ever smoker | 82/230 (35.7) | | 130/222 (58.6) | 59/134 (44.0) | 41/176 (23.3) | 108/255 (42.4) |  |
| Never smoker | 148/230 (64.3) | | 92/222 (41.4) | 75/134 (56.0) | 135/176 (76.7) | 147/255 (57.6) |  |
| Pack-year in smoker | 32.2 ± 19.2**^2^** | | 36.5 ± 21.9 | 34.6 ± 20.4 | 32.5 ± 17.9 | 36.2 ± 20.0 | 0.519 |
| Stage (n=1012)^3^ |  |  | |  |  |  | <0.001 |
| III | 54/232 (23.3) | 34/222 (15.3) | | 13/127 (10.2) | 37/176 (21.0) | 26/255 (10.2) |  |
| IV | 178/232 (76.7) | 188/222 (84.7) | | 114/127 (89.8) | 139/176 (79.0) | 229/255 (89.8) |  |
| FEV_1_ (n=706)^3^ | 76.3 ± 19.6 | 79.5 ± 19.5 | | 73.9 ± 21.2 | 83.0 ± 21.1 | 74.0 ± 18.6 | 0.001 |
| FVC (n=706)^3^ | 76.7 ± 17.2 | 81.9 ± 17.4 | | 78.3 ± 18.4 | 76.0 ± 16.7 | 76.1 ± 17.3 | 0.011 |
| CCI | 4.9 ± 1.5 | 6.9 ± 2.3 | | 4.6 ± 1.5 | 6.8 ± 2.3 | 5.4 ± 2.1 | <0.001 |
| Chemotherapy | 152 (65.2) | 214 (96.4) | | 81 (60.4) | 114 (64.8) | 202 (79.2) | <0.001 |
| TKI | 84 (36.1) | 139 (62.6) | | 39 (29.1) | 33 (18.8) | 120 (47.1) | <0.001 |
| Radiation therapy | 59 (25.3) | 125 (56.3) | | 23 (17.2) | 39 (22.2) | 26 (10.2) | <0.001 |
| Median OS**^4^** | 11.4 (9.3-13.6) | 17.3 (13.0-21.7) | | 13.4 (7.8-19.1) | 14.5 (10.6-18.4) | 15.4 (11.0-19.7) | 0.031 |
| Mean OS | 20.2 ± 1.5 | 27.6 ± 2.2 | | 23.8 ± 2.6 | 26.5 ± 2.8 | 25.4 ± 2.2 | 0.031 |

^1^ Number(%), ^2^Mean+/- SD, ^3^Differences in total number are due to missing values. **^4^**Median overall survival, month (95% confidence interval); Institute 1: Ewha Womans Univ.; 2: Inha Univ.; 3: Hallym Univ.; 4: Soonchunhyang Univ.; 5: Korea Univ.; BMI: body mass index, FEV_1_: forced expiratory volume in one second, FVC; forced vital capacity, CCI, Charlson comorbidity index, TKI: tyrosine kinase inhibitor.
