# Supplementary material for: Clinical significance of EGFR mutation types in lung adenocarcinoma: A multi-centre Korean study
Source: PLoS One. 2020 Feb 13;15(2):e0228925. doi: 10.1371/journal.pone.0228925 (PMC7018076; doi:10.1371/journal.pone.0228925)
Supplement: S3 Table — (DOCX) [file pone.0228925.s003.docx]

**S3 Table.** Univariate and multivariate Cox proportional hazard analysis for mortality in lung adenocarcinoma subjects

|  | **Univariate** | |  | **Multivariate model-1** | | |  | | **Multivariate model-2** | | | | |
| --- | --- | --- | --- | --- | --- | --- | --- | --- | --- | --- | --- | --- | --- |
|  | **HR (95% CI)** | **p-value** |  | **HR (95% CI)** | | **p-value** |  | | **HR (95% CI)** | | | **p-value** | |
| Presence of mutation^1^ | |  |  |  | |  |  | |  | | |  | |
| 18 | 0.916 (0.505-1.662) | 0.774 |  |  | |  |  | |  | | |  | |
| 19 | 0.487 (0.399-0.593) | <0.001 |  | 0.579 (0.470-0.714) | | <0.001 |  | |  | | |  | |
| 20 | 1.040 (0.573-1.886) | 0.898 |  |  | |  |  | |  | | |  | |
| 21 | 0.796 (0.654-0.970) | 0.024 |  |  | |  |  | | 1.020 (0.826-1.261) | | | 0.851 | |
| Age | 1.022 (1.015-1.029) | <0.001 |  | 1.018 (1.010-1.025) | | <0.001 |  | | 1.021 (1.013-1.028) | | | <0.001 | |
| Female | 0.696 (0.602-0.805) | <0.001 |  | 0.751 (0.646-0.872) | | <0.001 |  | | 0.729 (0.628-0.845) | | | <0.001 | |
| Low BMI | 1.717 (1.359-2.170) | <0.001 |  | 1.707 (1.347-2.162) | | <0.001 |  | | 1.643 (1.298-2.079) | | | <0.001 | |
| Never smoker | 0.797 (0.691-0.920) | 0.002 |  |  | |  |  | |  | | |  | |
| Stage IV | 1.395 (1.141-1.707) | 0.001 |  | 1.628 (1.324-2.001) | | <0.001 |  | | 1.641 (1.335-2.018) | | | <0.001 | |
| CCI | 1.093 (1.059-1.128) | <0.001 |  | 1.045 (1.008-1.083) | | 0.016 |  | | 1.037 (1.000-1.075) | | | 0.048 | |
| Chemotherapy | 0.698 (0.594-0.820) | <0.001 |  | 0.806 (0.668-0.972) | | 0.024 |  | |  | | |  | |
| TKI | 0.578 (0.499-0.668) | <0.001 |  | 0.699 (0.587-0.833) | | <0.001 |  | | 0.568 (0.489-0.660) | | | <0.001 | |
| Radiation therapy | 0.934 (0.798-1.094) | 0.398 |  |  | |  |  | |  | | |  | |
| Institute 4^2^ | 1 |  |  |  |  |  | |  | | | |  | |
| Institute 1 | 1.206 (0.970-1.500) | 0.092 |  |  |  |  | | | |  | |  |  |
| Institute 2 | 0.879 (0.704-1.098) | 0.257 |  |  |  |  | | | |  | |  |  |
| Institute 3 | 1.060 (0.818-1.372) | 0.661 |  |  |  |  | | | |  | |  |  |
| Institute 5 | 0.927 (0.744-1.156) | 0.503 |  |  |  |  | | | |  |  | |  |

^1^Reference: EGFR negative; ^2^Reference: Institute 4. Institute 4 had survival duration which the closest to the median and mean overall survival among 5 institutes. BMI, body mass index; CCI, Charlson comorbidity index; TKI, tyrosine kinase inhibitor
