# Supplementary material for: Clinical significance of EGFR mutation types in lung adenocarcinoma: A multi-centre Korean study
Source: PLoS One. 2020 Feb 13;15(2):e0228925. doi: 10.1371/journal.pone.0228925 (PMC7018076; doi:10.1371/journal.pone.0228925)
Supplement: S4 Table — (DOCX) [file pone.0228925.s004.docx]

**S4 Table. Comparison of baseline characteristics between exon 19 deletions and exon 18/20/21 mutations in EGFR positive lung adenocarcinoma subjects**

|  | **19 deletions** | **18/20/21 mutations** | **p-value** |
| --- | --- | --- | --- |
| Number (n) | 198 | 190 |  |
| Age | 64.1 ± 11.9^1^ | 68.5 ± 10.5^1^ | <0.001 |
| Sex |  |  | 0.932 |
| Male | 79 (39.9) ^2^ | 75 (39.5)^2^ |  |
| Female | 119 (60.1) | 115 (60.5) |  |
| Low BMI (<18.5 kg/m^2^) | 17 (8.6) | 11 (5.8) | 0.287 |
| Smoking status (n=387)^3^ |  |  | 0.930 |
| Ever smoker | 50/198 (25.3) | 47/189 (24.9) |  |
| Never smoker | 148/198 (74.7) | 142/189 (75.1) |  |
| Smoking amount in smoker, pack-years | 27.8 ± 16.9^b^ | 28.2 ± 19.6 | 0.915 |
| Stage (n=386)^3^ |  |  | 0.986 |
| III | 22/197 (11.2) | 21/189 (11.1) |  |
| IV | 175/197 (88.8) | 168/189 (88.9) |  |
| Treatment |  |  |  |
| Chemotherapy | 157 (79.3) | 164 (86.3) | 0.067 |
| TKI | 140 (70.7) | 144 (75.8) | 0.259 |
| Radiation therapy | 56 (28.3) | 50 (26.3) | 0.664 |
| FEV_1_ (n=269)^3^ | 79.0 ± 17.8 | 79.5 ± 21.1 | 0.819 |
| FVC (n=269)^3^ | 76.9 ± 16.8 | 78.4 ± 18.6 | 0.494 |
| CCI | 5.5 ± 2.2 | 5.7 ± 2.2 | 0.496 |
| Median overall survival, month (95% CI) | 29.9 (22.4-37.4) | 19.2 (16.1-22.4) | <0.001 |

^1^Mean±SD, ^2^Number (%), ^3^Differences in total number are due to missing values. BMI, body mass index; TKI, tyrosine kinase inhibitor; FEV_1_, forced expiratory volume in 1 second; FVC, forced vital capacity; CCI, Charlson comorbidity index; CI, confidence interval.
