# Supplementary figures and images for: Clinical significance of EGFR mutation types in lung adenocarcinoma: A multi-centre Korean study
Source: PLoS One. 2020 Feb 13;15(2):e0228925. doi: 10.1371/journal.pone.0228925 (PMC7018076; doi:10.1371/journal.pone.0228925)

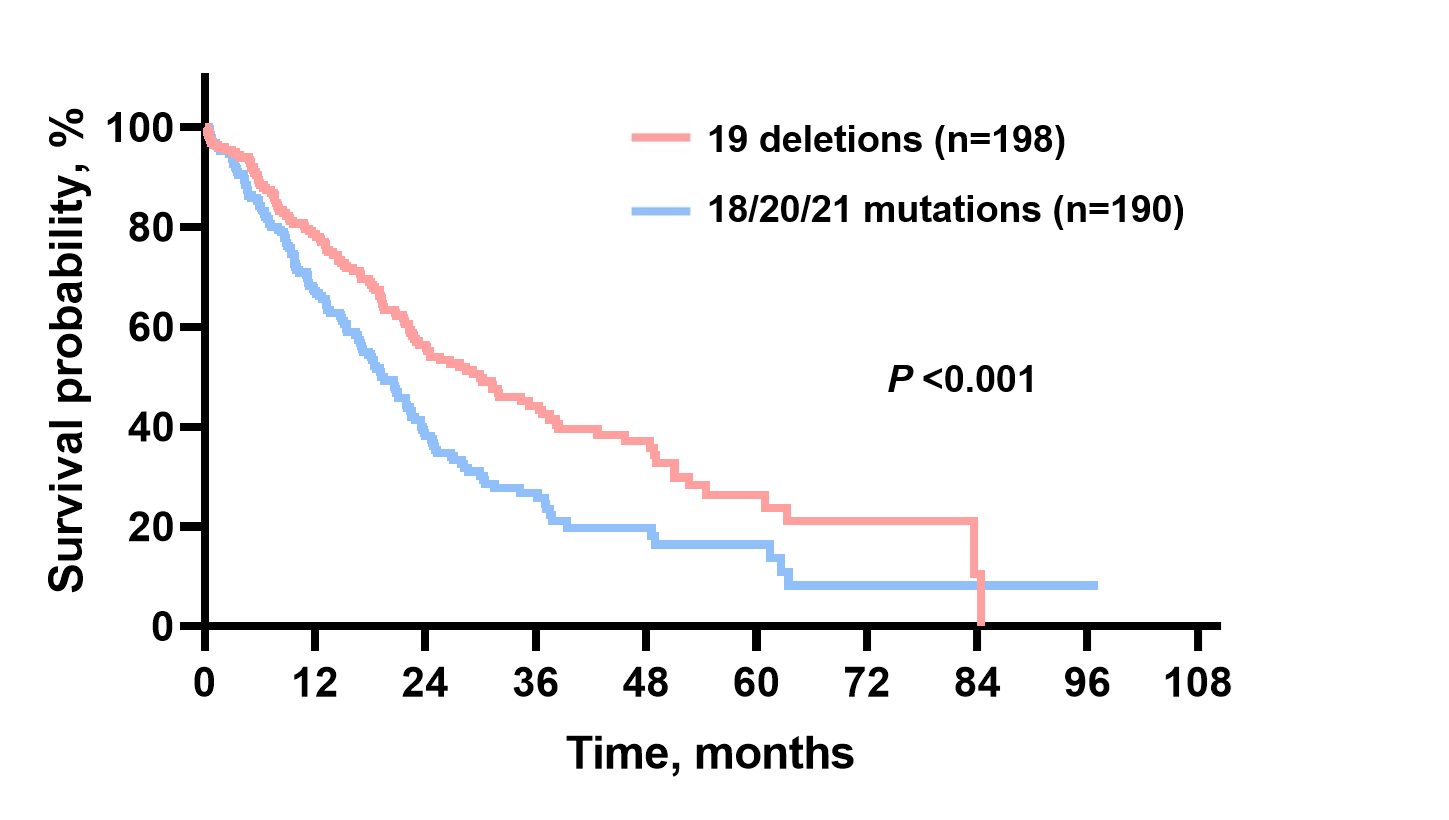

Supplement: S1 Fig — (TIF) [file pone.0228925.s005.tif]
